# A Glaucoma Patient with an Intraocular Pressure Decrease following Total Gastrectomy and Postoperative Anticancer Treatment

**DOI:** 10.1155/2023/9529229

**Published:** 2023-02-14

**Authors:** Yukihisa Takada, Takayoshi Sumioka, Tadahiko Tamura, Shizuya Saika

**Affiliations:** Department of Ophthalmology, School of Medicine, Wakayama Medical University, 811-1 Kimiidera, Wakayama 641-0012, Japan

## Abstract

We herein report a case of glaucoma with an intraocular pressure (IOP) decrease following total gastrectomy (TG) and anticancer treatment for gastric cancer. A 62-year-old male underwent trabeculectomy of the left and right eyes in August 2011 and July 2012, respectively. During the follow-up, IOP of the right eye was 9-12 mmHg (with bimatoprost, dorzolamide, and timolol maleate), and that of the left eye ranged between 14 and 26 mmHg (with bimatoprost, dorzolamide, timolol maleate, and brimonidine tartrate). In December 2014, TG was performed due to gastric cancer. After surgery, the patient received S-1+CDDP, weekly PAC, and CPT-11 therapies. The patient died on March X, 2017. Before TG, the body mass index (BMI) was 29.5 but decreased to 24.8 before the start of the two courses of weekly PAC therapy. IOP of the right eye was 6 mmHg (with bimatoprost), and that of the left eye was 10 mmHg (with bimatoprost, dorzolamide, and brimonidine tartrate), showing decreases. After the initiation of weekly PAC therapy, BMI was approximately 19. IOP of the right eye ranged between 6 and 10 mmHg until the final ophthalmological examination (January 11, 2017), while that of the left eye ranged between 8 and 15 mmHg. In this patient with glaucoma, IOP was not controlled by eye drop treatment, and TG for gastric cancer and postoperative treatment with anticancer drugs resulted in weight loss and a decrease in IOP.

## 1. Introduction

Even when glaucoma treatment with several eye drop preparations is performed, a target intraocular pressure cannot be achieved in some cases. Diurnal changes [1], seasons [2], and posture [3] have been identified as temporary or reversible factors affecting intraocular pressure and aging [4], diabetes mellitus [5] as a systemic disease, and steroid therapy [6] as long-term factors.

Previous studies reported reductions in intraocular pressure following radical prostatectomy for prostatic cancer [7] and total gastrectomy to achieve weight loss for obese patients [8, 9], suggesting a relationship between weight loss and a decrease in intraocular pressure. However, the effects of total gastrectomy for gastric cancer on intraocular pressure remain unknown. Although anticancer drugs have been shown to induce various ocular complications, such as corneal disorder, uveitis, and cystoid macular edema, it currently remains unclear whether they affect intraocular pressure.

We herein present a case of glaucoma in which intraocular pressure decreased after total gastrectomy for gastric cancer and postoperative treatment with anticancer drugs.

## 2. Case Presentation

*Patient*: a 62-year-old male

*Medical history*: not contributory

*Ophthalmological history*: due to primary open angle glaucoma, trabeculectomy of the left eye was performed in Wakayama Medical University Hospital (our hospital) in August 2011. The same procedure was conducted on the right eye in July 2012

*Present illness*: after glaucoma surgery (right eye) in July 2012, treatment was continued at a local clinic; however, the patient was referred to our hospital for subsequent glaucoma control on October X, 2012

### 2.1. Findings of the Initial Examination in Our Department Clinic

*Right eye*: eleven o'clock basal iridectomy. Diffuse filtering blebs of the epiotic conjunctiva, cataract, and excavation/enlargement of the optic nerve head were noted

*Left eye*: one o'clock basal iridectomy. Diffuse filtering blebs of the epiotic conjunctiva, cataract, and excavation/enlargement of the optic nerve head were observed

Visual acuity of the right eye was 0.15 (1.0), and that of the left eye was 0.06 (0.4). Intraocular pressure measured using the Goldmann applanation tonometry was 7 mmHg in the right eye and 17 mmHg in the left eye. On static perimetry (Humphrey 30-2) after glaucoma surgery for the right eye in August 2012, the MD value of the right eye was -8.53 dB and that of the left eye was -29.40 dB.

### 2.2. Use of Eye Drops

*Right eye*: none

*Left eye*: bimatoprost, dorzolamide, and timolol maleate

**Figure 1:**
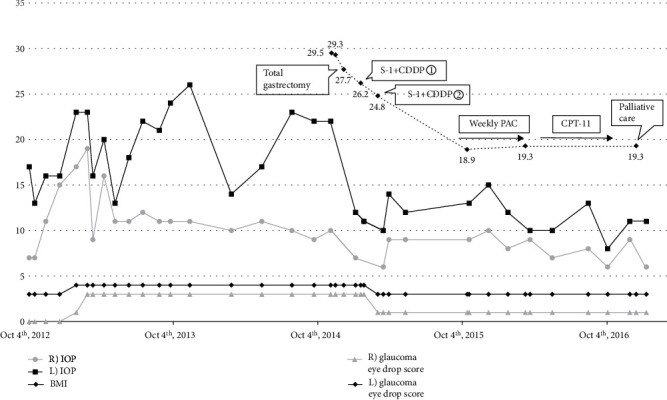
The course of changes in intraocular pressure, body mass index, and glaucoma eye drop score. Vertical axis: mmHg (intraocular pressure), points (glaucoma eye drop score), and body mass index.

Changes in intraocular pressure, body mass index (BMI), and glaucoma eye drop scores are shown in Figure 1.

### 2.3. Clinical Course

On January X, 2013, intraocular pressure increased in the bilateral eyes (right: 17 mmHg and left: 23 mmHg). Intraocular pressure was hereafter measured using the Goldmann applanation tonometry. The patient did not wish for additional treatment with invasiveness, such as needling, because the right eye was the superior eye and the only eye remaining intact. After eye drop potentiation (right eye: bimatoprost, dorzolamide, and timolol maleate and left eye: brimonidine tartrate), right intraocular pressure was stable at 9-12 mmHg. However, left intraocular pressure ranged between 14 and 26 mmHg and was unstable. Although additional glaucoma surgery was proposed, a follow-up only was continued based on the patient's wishes. On February X, 2013, visual acuity of the right eye was 0.2 (1.0), and that of the left eye was 0.04 (0.2). Intraocular pressure was 9 mmHg in the right eye and 16 mmHg in the left eye. On static perimetry (Humphrey 30-2), the MD value of the right eye was -12.05 dB, and that of the left eye was -29.46 dB.

On December 15, 2014, surgery (laparoscopic total gastrectomy/right colectomy and D2 lymph node dissection) for gastric and ascending colon cancers was performed. Before surgery (November 5), intraocular pressure of the right eye was 10 mmHg, and that of the left eye was 22 mmHg.

The patient received two courses of S-1+cisplatin (CDDP) therapy as a general regimen for gastric cancer from January 20, 2015. At the start of the 1^st^ course, BMI was 26.2 (January 20), and right and left intraocular pressures were 7 and 12 mmHg, respectively (January 7). Therefore, dorzolamide in the right eye and timolol maleate in both eyes were discontinued. At the start of the 2^nd^ course, BMI was 24.8 (March 4), and right and left intraocular pressures were 6 and 10 mmHg, respectively (March 18). Due to an infection caused by febrile neutropenia, S-1+CDDP therapy was completed in 2 courses.

Weekly paclitaxel (PAC) therapy was performed to reduce neutropenia from October 16, 2015, until April 8, 2016 (BMI on October 16: 18.9 and BMI on March 11, 2016: 19.3). On October X, 2015, visual acuity of the right eye was 0.07 (1.0), and that of the left eye was 0.01 (0.01). Intraocular pressure of the right eye was 10 mmHg, and that of the left eye was 16 mmHg. On static perimetry (Humphrey 30-2), the MD value of the right eye was -10.72 dB, and that of the left eye was -31.02 dB. On August 2, 2016, recurrent ascending colon cancer and multiple metastases (liver and lung) were detected. Irinotecan (CPT-11) therapy was administered as anticancer therapy between August 19 and December 9. The exacerbation of ascites was noted on December 11, and the general condition of the patient deteriorated. On December 16, the patient was admitted to the palliative care unit (BMI: 19.3). During anticancer therapy, there were no changes to eye drop preparations for glaucoma. Right intraocular pressure ranged between 6 and 10 mmHg and left intraocular pressure between 8 and 15 mmHg. On May X, 2016, visual acuity of the right eye was 0.06 (0.8), and that of the left eye was 0.01 (0.01). Intraocular pressure of the right eye was 7 mmHg, and that of the left eye was 10 mmHg. On static perimetry (Humphrey 30-2), the MD value of the right eye was -7.85 dB, and that of the left eye was -30.93 dB. The date of the final ophthalmological consultation after admission to the palliative care unit was January 11, 2017 (right intraocular pressure: 6 mmHg and left intraocular pressure: 11 mmHg). The patient died on March X, 2017.

## 3. Discussion

This case report was conducted in accordance with the Declaration of Helsinki and its amendments or comparable ethical standards.

Posarelli et al. reported that gastric bypass for obesity significantly decreased BMI but did not significantly affect intraocular pressure [10]. However, other studies indicated that gastrectomy for obesity significantly decreased BMI and intraocular pressure [8, 9], suggesting a relationship between body weight changes related to gastrectomy for obesity and changes in intraocular pressure.

However, there is currently no information on changes in intraocular pressure following gastrectomy regardless of a history of glaucoma surgery.

In the present case, BMI before total gastrectomy was 29.3, that on the 1^st^ course of postoperative S-1+CDDP therapy following total gastrectomy was 26.2, and that on the 2^nd^ course was 24.8. At the start of weekly PAC therapy, BMI was 18.9, showing a decrease. BMI was approximately 19 from the administration of CPT-11 until the start of palliative care as terminal care, during which the final body weight measurement was conducted. According to the WHO criteria for obesity, BMI before total gastrectomy corresponded to “preobesity (BMI: 25.00-29.99)” and that after the 2^nd^ course of S-1+CDDP therapy to “a normal body weight (BMI: 18.50-24.99).” BMI on glaucoma surgery for the left eye in August 2011 was 31.1, and that on glaucoma surgery for the right eye in July 2012 was 28.6.

Chemotherapy may have increased weight loss or decreased intraocular pressure. In the present case, S-1, PAC, CDDP, and CPT-11were used for anticancer treatment. Keratoconjunctivitis [11], macular edema [12], and neuropathy [13] have been reported as representative adverse ocular reactions to S-1, PAC, and CDDP, respectively. However, the relationship between anticancer drugs and intraocular pressure decreases remains unknown. There have been no case reports of adverse ocular reactions to CPT-11. Nausea, vomiting, diarrhea, and anorexia are observed as adverse reactions to anticancer drugs; therefore, the above anticancer drugs may have contributed to weight loss after total gastrectomy in the present case and may have been indirectly associated with the decrease observed in intraocular pressure.

Regarding changes in intraocular pressure from surgery for gastric cancer and anticancer therapy until palliative care, intraocular pressure of the right eye before total gastrectomy was 10 mmHg (glaucoma eye drop score: 3 points. The glaucoma eye drop score was 1 point for 1 ingredient of the glaucoma eye drop and 2 points for the oral administration of acetazolamide). However, as shown in the figure, it decreased after total gastrectomy to between 6 and 10 mmHg (glaucoma eye drop score: 1 point) from anticancer therapy to the final intraocular pressure measurement. Intraocular pressure of the left eye before total gastrectomy was 22 mmHg (glaucoma eye drop score: 4 points) but decreased after total gastrectomy, ranging between 8 and 15 mmHg (glaucoma eye drop score: 3 points) from anticancer therapy to the final intraocular pressure measurement. After the initiation of gastric cancer treatment, BMI began to decrease. There were also simultaneous decreases in the number of eye drops for glaucoma and intraocular pressure.

Burgansky-Eliash et al. reported that an increase in episcleral venous pressure and choroidal vessel congestion due to a high intraperitoneal pressure elevated intraocular pressure in obese patients, suggesting that intraperitoneal pressure decreases with weight loss, thereby reducing intraocular pressure through a reduction in episcleral venous pressure and an improvement in choroidal vessel blood flow [8]. There is currently no information on changes in intraocular pressure before and after radical total gastrectomy for gastric cancer. However, in the present case, weight loss as a result rather than the purpose of surgery or the effects of gastrectomy itself may have led to the decrease observed in intraocular pressure. Furthermore, a reduction in external pressure related to a weight loss-related decrease in the intraorbital adipose tissue volume may have contributed to the lower intraocular pressure. However, the rate of decrease in BMI from total gastrectomy until the initiation of S-1+CDDP therapy was small (29.3→26.2), whereas that in intraocular pressure was large (right eye: 10→7 mmHg and left eye: 22→12 mmHg); therefore, total gastrectomy may have induced a decrease in intraocular pressure.

In the future, the presence or absence of body weight changes after total gastrectomy for gastric cancer, and changes in intraocular pressure need to be examined in a large-scale study.
